# Supplementary material for: Screening Leads to Overestimated Associations of Thyroid Dysfunction and Thyroiditis with Thyroid Cancer Risk
Source: Cancers (Basel). 2021 Oct 27;13(21):5385. doi: 10.3390/cancers13215385 (PMC8582587; doi:10.3390/cancers13215385)
Supplement: Supplementary file 1 [file cancers-13-05385-s001.zip › cancers-1428543-supplementary.pdf]

Supplemental Materials

Screening Leads to Overestimated Associations of Thyroid Dysfunction and Thyroiditis with Thyroid Cancer Risk

Young Shin Song [1], Kyung Soo Kim [1], Soo Kyung Kim [1], Young Wook Cho [1] and Hyo Geun Choi [2,3,*]

Table S1. Subgroup analyses according to age, sex, income, and region of residence in each thyroid disease calculating odds ratios (95% confidence intervals) for thyroid cancer in Study I.

Characteristics	Thyroid cancer (exposure/total, %)	Control (exposure/total, %)	Odds ratios for thyroid cancer							
			Crude†	*P*-value	Model 1†‡	*P*-value	Model 2†§	*P*-value	Model 3†‖	*P*-value
Age <60 years old (n = 19,020)										
Hypothyroidism	477/3,804 (12.5)	603/15,216 (4.0)	3.53 (3.11-4.00)	<0.001*	3.59 (3.15-4.09)	<0.001*	2.98 (2.60-3.42)	<0.001*	3.43 (2.99-3.92)	<0.001*
Hyperthyroidism	251/3,804 (6.6)	433/15,216 (2.9)	2.42 (2.06-2.83)	<0.001*	2.41 (2.04-2.84)	<0.001*	1.78 (1.49-2.12)	<0.001*	N/A	
Thyroiditis	233/3,804 (6.1)	308/15,216 (2.0)	3.19 (2.68-3.80)	<0.001*	3.06 (2.55-3.67)	<0.001*	1.95 (1.60-2.37)	<0.001*	N/A	
Autoimmune thyroiditis	97/3,804 (2.6)	142/15,216 (0.9)	2.79 (2.15-3.62)	<0.001*	2.66 (2.02-3.49)	<0.001*	N/A		1.49 (1.12-1.98)	0.007*
Graves' disease	27/3,804 (0.7)	53/15,216 (0.4)	2.04 (1.28-3.25)	0.003*	1.98 (1.21-3.22)	0.006*	N/A		1.65 (0.99-2.74)	0.054
Age ≥60 years old (n = 9,310)										
Hypothyroidism	233/1,862 (12.5)	280/7,448 (3.8)	3.72 (3.09-4.46)	<0.001*	3.79 (3.14-4.57)	<0.001*	3.23 (2.66-3.93)	<0.001*	3.54 (2.92-4.28)	<0.001*

Hyperthyroidism	118/1,862 (6.3)	229/7,448 (3.1)	2.14 (1.70-2.69)	<0.001*	2.16 (1.71-2.74)	<0.001*	1.66 (1.30-2.13)	<0.001*	N/A	
Thyroiditis	106/1,862 (5.7)	137/7,448 (1.8)	3.25 (2.51-4.22)	<0.001*	3.40 (2.60-4.44)	<0.001*	2.53 (1.91-3.34)	<0.001*	N/A	
Autoimmune thyroiditis	53/1,862 (2.9)	63/7,448 (0.9)	3.45 (2.38-5.00)	<0.001*	3.61 (2.47-5.27)	<0.001*	N/A		2.57 (1.73-3.83)	<0.001*
Graves' disease	14/1,862 (0.8)	24/7,448 (0.3)	2.35 (1.21-4.55)	0.012*	2.51 (1.28-4.90)	0.007*	N/A		2.02 (1.00-4.10)	0.052
Males (n = 5,980)										
Hypothyroidism	81/1,196 (6.8)	32/4,784 (0.7)	10.69 (7.07-16.17)	<0.001*	10.58 (6.88-16.27)	<0.001*	8.63 (5.55-13.43)	<0.001*	10.39 (6.73-16.05)	<0.001*
Hyperthyroidism	61/1,196 (5.1)	70/4,784 (1.5)	3.62 (2.56-5.14)	<0.001*	3.65 (2.53-5.28)	<0.001*	2.52 (1.69-3.77)	<0.001*	N/A	
Thyroiditis	25/1,196 (2.1)	17/4,784 (0.4)	6.00 (3.23-11.14)	<0.001*	6.24 (3.30-11.80)	<0.001*	3.45 (1.72-6.92)	0.001*	N/A	
Autoimmune thyroiditis	7/1,196 (0.6)	11/4,784 (0.2)	2.55 (0.99-6.59)	0.053	2.82 (1.08-7.36)	0.034*	N/A		1.23 (0.39-3.88)	0.723
Graves' disease	5/1,196 (0.4)	8/4,784 (0.2)	2.50 (0.82-7.66)	0.108	2.29 (0.70-7.49)	0.172	N/A		1.45 (0.38-5.55)	0.590
Females (n = 22,350)										
Hypothyroidism	629/4,470 (14.1)	851/17,880 (4.8)	3.29 (2.95-3.66)	<0.001*	3.37 (3.02-3.77)	<0.001*	2.84 (2.52-3.19)	<0.001*	3.16 (2.82-3.55)	<0.001*
Hyperthyroidism	308/4,470 (6.9)	592/17,880 (3.3)	2.16 (1.88-2.49)	<0.001*	2.17 (1.87-2.51)	<0.001*	1.62 (1.39-1.89)	<0.001*	N/A	
Thyroiditis	314/4,470 (7.0)	428/17,880 (2.4)	3.09 (2.66-3.59)	<0.001*	3.05 (2.61-3.56)	<0.001*	2.09 (1.78-2.46)	<0.001*	N/A	
Autoimmune thyroiditis	143/4,470 (3.2)	194/17,880 (1.1)	3.02 (2.43-3.76)	<0.001*	2.97 (2.37-3.73)	<0.001*	N/A		1.86 (1.47-2.36)	<0.001*
Graves' disease	36/4,470 (0.8)	69/17,880 (0.4)	2.10 (1.40-3.14)	<0.001*	2.15 (1.42-3.26)	<0.001*	N/A		1.81 (1.17-2.78)	0.007*
Low income (n = 11,215)										

Hypothyroidism	293/2,243 (13.1)	330/8,972 (3.7)	3.97 (3.37-4.69)	<0.001*	4.00 (3.37-4.74)	<0.001*	3.31 (2.77-3.96)	<0.001*	3.76 (3.16-4.48)	<0.001*
Hyperthyroidism	144/2,243 (6.4)	262/8,972 (2.9)	2.28 (1.85-2.81)	<0.001*	2.35 (1.90-2.92)	<0.001*	1.68 (1.34-2.12)	<0.001*	N/A	
Thyroiditis	142/2,243 (6.3)	171/8,972 (1.9)	3.50 (2.79-4.40)	<0.001*	3.44 (2.72-4.36)	<0.001*	2.25 (1.75-2.90)	<0.001*	N/A	
Autoimmune thyroiditis	61/2,243 (2.7)	79/8,972 (0.9)	3.16 (2.25-4.43)	<0.001*	3.10 (2.19-4.40)	<0.001*	N/A		1.79 (1.23-2.60)	0.002*
Graves' disease	19/2,243 (0.9)	29/8,972 (0.3)	2.63 (1.47-4.70)	0.001*	2.70 (1.48-4.91)	0.001*	N/A		2.18 (1.17-4.07)	0.015*
High income (n = 17,115)										
Hypothyroidism	417/3,423 (12.2)	553/13,692 (4.0)	3.35 (2.93-3.84)	<0.001*	3.45 (3.01-3.96)	<0.001*	2.90 (2.51-3.35)	<0.001*	3.25 (2.82-3.75)	<0.001*
Hyperthyroidism	225/3,423 (6.6)	400/13,692 (2.9)	2.34 (1.98-2.77)	<0.001*	2.31 (1.94-2.75)	<0.001*	1.77 (1.47-2.12)	<0.001*	N/A	
Thyroiditis	197/3,423 (5.8)	274/13,692 (2.0)	3.02 (2.50-3.65)	<0.001*	3.02 (2.49-3.67)	<0.001*	2.05 (1.67-2.52)	<0.001*	N/A	
Autoimmune thyroiditis	89/3,423 (2.6)	126/13,692 (0.9)	2.89 (2.20-3.80)	<0.001*	2.91 (2.19-3.86)	<0.001*	N/A		1.81 (1.34-2.43)	<0.001*
Graves' disease	22/3,423 (0.6)	48/13,692 (0.4)	1.84 (1.11-3.05)	0.018*	1.84 (1.09-3.10)	0.022*	N/A		1.52 (0.88-2.62)	0.137
Urban (n = 13,595)										
Hypothyroidism	355/2,719 (13.1)	470/10,876 (4.3)	3.37 (2.91-3.90)	<0.001*	3.38 (2.90-3.93)	<0.001*	2.84 (2.43-3.33)	<0.001*	3.18 (2.72-3.71)	<0.001*
Hyperthyroidism	172/2,719 (6.3)	317/10,876 (2.9)	2.25 (1.86-2.73)	<0.001*	2.23 (1.83-2.72)	<0.001*	1.63 (1.32-2.00)	<0.001*	N/A	
Thyroiditis	178/2,719 (6.6)	258/10,876 (2.4)	2.91 (2.39-3.54)	<0.001*	2.81 (2.29-3.45)	<0.001*	1.91 (1.54-2.37)	<0.001*	N/A	
Autoimmune thyroiditis	83/2,719 (3.1)	124/10,876 (1.1)	2.74 (2.07-3.64)	<0.001*	2.69 (2.01-3.60)	<0.001*	N/A		1.65 (1.21-2.24)	0.002*
Graves' disease	21/2,719 (0.8)	37/10,876 (0.3)	2.28 (1.33-3.90)	0.003*	2.32 (1.34-4.04)	0.003*	N/A		1.86 (1.04-3.31)	0.036*

Rural (n = 14,735)										
Hypothyroidism	355/2,947 (12.1)	413/11,788 (3.5)	3.83 (3.30-4.45)	<0.001*	3.96 (3.40-4.62)	<0.001*	3.29 (2.81-3.86)	<0.001*	3.74 (3.20-4.37)	<0.001*
Hyperthyroidism	197/2,947 (6.7)	345/11,788 (2.9)	2.38 (1.99-2.85)	<0.001*	2.41 (2.00-2.90)	<0.001*	1.83 (1.51-2.23)	<0.001*	N/A	
Thyroiditis	161/2,947 (5.5)	187/11,788 (1.6)	3.61 (2.91-4.48)	<0.001*	3.67 (2.94-4.58)	<0.001*	2.43 (1.92-3.08)	<0.001*	N/A	
Autoimmune thyroiditis	67/2,947 (2.3)	81/11,788 (0.7)	3.37 (2.43-4.67)	<0.001*	3.38 (2.41-4.73)	<0.001*	N/A		2.04 (1.43-2.91)	<0.001*
Graves' disease	20/2,947 (0.7)	40/11,788 (0.3)	2.01 (1.17-3.44)	0.011*	2.00 (1.14-3.50)	0.016*	N/A		1.68 (0.94-3.01)	0.081

* Conditional logistic regression, Significance at $P < 0.05$, † Models were stratified by age, sex, income, and region of residence. ‡ Model 1 was adjusted for total cholesterol, systolic blood pressure, diastolic blood pressure, fasting blood glucose, obesity, smoking, alcohol consumption, and Charlson comorbidity index scores. § Model 2 was adjusted for model 1 plus hypothyroidism, hyperthyroidism, and thyroiditis. ‖ Model 3 was adjusted for model 1 plus hypothyroidism, autoimmune thyroiditis, and Graves' disease.

Table S2. Subgroup analyses according to thyroid diseases in each thyroid disease calculating odds ratios (95% confidence intervals) for thyroid cancer in Study I.

Characteristics	Thyroid cancer	Control	Odds ratios for thyroid cancer							
	(exposure/total, %)	(exposure/total, %)	Crude	*P*-value	Model 1†	*P*-value	Model 2‡	*P*-value	Model 3§	*P*-value
Non-hypothyroidism (n = 26,737)										
Hypothyroidism	0/4,956 (0.0)	0/21,781 (0.0)	N/A		N/A		N/A		N/A	
Hyperthyroidism	264/4,956 (5.3)	516/21,781 (2.4)	2.33 (2.00-2.71)	<0.001*	2.37 (2.02-2.77)	<0.001*	2.14 (1.82-2.51)	<0.001*	N/A	
Thyroiditis	232/4,956 (4.7)	274/21,781 (1.3)	3.91 (3.27-4.67)	<0.001*	3.82 (3.18-4.60)	<0.001*	3.51 (2.91-4.23)	<0.001*	N/A	
Autoimmune thyroiditis	98/4,956 (2.0)	98/21,781 (0.5)	4.51 (3.40-5.98)	<0.001*	4.29 (3.20-5.75)	<0.001*	N/A		4.24 (3.16-5.69)	<0.001*
Graves' disease	31/4,956 (0.6)	64/21,781 (0.3)	2.15 (1.40-3.30)	0.001*	2.20 (1.41-3.44)	0.001*	N/A		2.08 (1.32-3.28)	0.002*
Hypothyroidism (n= 1,593)										
Hypothyroidism	710/710 (100.0)	883/883 (100.0)	N/A		N/A		N/A		N/A	

Hyperthyroidism	105/710 (14.8)	146/883 (16.5)	0.84 (0.64-1.12)	0.233	0.81 (0.60-1.08)	0.146	0.82 (0.61-1.09)	0.170	N/A	
Thyroiditis	107/710 (15.1)	171/883 (19.4)	0.77 (0.59-1.01)	0.060	0.78 (0.59-1.04)	0.086	0.79 (0.60-1.05)	0.099	N/A	
Autoimmune thyroiditis	52/710 (7.3)	107/883 (12.1)	0.60 (0.42-0.85)	0.004*	0.62 (0.43-0.89)	0.009*	N/A		0.62 (0.43-0.89)	0.009*
Graves’ disease	10/710 (1.4)	13/883 (1.5)	0.89 (0.38-2.07)	0.783	0.78 (0.32-1.89)	0.576	N/A		0.78 (0.32-1.90)	0.585
Non-hyperthyroidism (n = 27,299)										
Hypothyroidism	605/5,297 (11.4)	737/22,002 (3.4)	3.77 (3.37-4.22)	<0.001*	3.88 (3.46-4.36)	<0.001*	3.40 (3.01-3.83)	<0.001*	3.62 (3.22-4.08)	<0.001*
Hyperthyroidism	0/5,297 (0.0)	0/22,002 (0.0)	N/A		N/A		N/A		N/A	
Thyroiditis	294/5,297 (5.6)	360/22,002 (1.6)	3.56 (3.04-4.17)	<0.001*	3.60 (3.06-4.24)	<0.001*	2.53 (2.13-3.00)	<0.001*	N/A	
Autoimmune thyroiditis	133/5,297 (2.5)	151/22,002 (0.7)	3.74 (2.96-4.74)	<0.001*	3.77 (2.96-4.81)	<0.001*	N/A		2.33 (1.80-3.01)	<0.001*
Graves’ disease	0/5,297 (0.0)	0/22,002 (0.0)	N/A		N/A		N/A		N/A	
Hyperthyroidism (n = 1,031)										
Hypothyroidism	105/369 (28.5)	146/662 (22.1)	1.46 (1.09-1.96)	0.012*	1.45 (1.06-1.97)	0.019*	1.49 (1.09-2.03)	0.013*	1.57 (1.14-2.15)	0.005*
Hyperthyroidism	369/369 (100.0)	662/662 (100.0)	N/A		N/A		N/A		N/A	
Thyroiditis	45/369 (12.2)	85/662 (12.8)	0.94 (0.64-1.39)	0.771	0.87 (0.57-1.31)	0.490	0.80 (0.53-1.22)	0.298	N/A	
Autoimmune thyroiditis	17/369 (4.6)	54/662 (8.2)	0.55 (0.31-0.97)	0.038*	0.50 (0.28-0.92)	0.025*	N/A		0.44 (0.24-0.81)	0.008*
Graves’ disease	41/369 (11.1)	77/662 (11.6)	0.94 (0.63-1.41)	0.775	0.92 (0.60-1.41)	0.701	N/A		0.95 (0.62-1.45)	0.799
Non-Graves’ disease (n = 28,212)										
Hypothyroidism	700/5,625 (12.4)	870/22,587 (3.9)	3.60 (3.24-4.00)	<0.001*	3.69 (3.31-4.11)	<0.001*	3.08 (2.75-3.45)	<0.001*	3.47 (3.11-3.88)	<0.001*
Hyperthyroidism	328/5,625 (5.8)	585/22,587 (2.6)	2.33 (2.03-2.68)	<0.001*	2.35 (2.04-2.71)	<0.001*	1.73 (1.49-2.01)	<0.001*	N/A	

Thyroiditis	334/5,625 (5.9)	436/22,587 (1.9)	3.23 (2.79-3.74)	<0.001*	3.25 (2.79-3.78)	<0.001*	2.17 (1.85-2.55)	<0.001*	N/A	
Autoimmune thyroiditis	149/5,625 (2.7)	198/22,587 (0.9)	3.09 (2.49-3.83)	<0.001*	3.12 (2.50-3.89)	<0.001*	N/A		1.89 (1.50-2.39)	<0.001*
Graves' disease	0/5,625 (0.0)	0/22,587 (0.0)	N/A		N/A		N/A		N/A	
Graves' disease (n = 118)										
Hypothyroidism	10/41 (24.4)	13/77 (16.9)	1.61 (0.63-4.11)	0.322	1.46 (0.52-4.14)	0.477	1.52 (0.53-4.36)	0.438	1.63 (0.53-5.02)	0.396
Hyperthyroidism	41/41 (100.0)	77/77 (100.0)	N/A		N/A		N/A		N/A	
Thyroiditis	5/41 (12.2)	9/77 (11.7)	1.00 (0.31-3.23)	0.994	0.55 (0.13-2.30)	0.412	0.53 (0.13-2.22)	0.383	N/A	
Autoimmune thyroiditis	1/41 (2.4)	7/77 (9.1)	0.21 (0.03-1.85)	0.161	0.04 (0.00-0.73)	0.031*	N/A		0.04 (0.002-0.69)	0.027*
Graves' disease	41/41 (100.0)	77/77 (100.0)	N/A		N/A		N/A		N/A	
Non-thyroiditis (n = 27,546)										
Hypothyroidism	603/5,327 (11.3)	712/22,219 (3.2)	3.92 (3.50-4.39)	<0.001*	3.99 (3.55-4.48)	<0.001*	3.99 (3.55-4.48)	<0.001*	3.96 (3.52-4.45)	<0.001*
Hyperthyroidism	324/5,327 (6.1)	577/22,219 (2.6)	2.44 (2.12-2.81)	<0.001*	2.48 (2.15-2.87)	<0.001*	2.48 (2.15-2.87)	<0.001*	N/A	
Thyroiditis	0/5,327 (0.0)	0/22,219 (0.0)	N/A		N/A		N/A		N/A	
Autoimmune thyroiditis	0/5,327 (0.0)	0/22,219 (0.0)	N/A		N/A		N/A		N/A	
Graves' disease	36/5,327 (0.7)	68/22,219 (0.3)	2.23 (1.48-3.34)	<0.001*	2.35 (1.55-3.55)	<0.001*	N/A		1.96 (1.27-3.02)	0.002*
Thyroiditis (n = 784)										
Hypothyroidism	107/339 (31.6)	171/445 (38.4)	0.75 (0.56-1.02)	0.063	0.74 (0.53-1.01)	0.061	0.75 (0.55-1.04)	0.085	0.73 (0.53-1.01)	0.060
Hyperthyroidism	45/339 (13.3)	85/445 (19.1)	0.61 (0.41-0.90)	0.014*	0.58 (0.38-0.88)	0.011*	0.59 (0.39-0.90)	0.015*	N/A	
Thyroiditis	339/339 (100.0)	445/445 (100.0)	N/A		N/A		N/A		N/A	

Autoimmune thyroiditis	150/339 (44.3)	205/445 (46.1)	0.95 (0.71-1.27)	0.724	0.96 (0.71-1.29)	0.764	N/A		1.02 (0.75-1.39)	0.913
Graves' disease	5/339 (1.5)	9/445 (2.0)	0.66 (0.22-2.01)	0.464	0.40 (0.11-1.40)	0.151	N/A		0.39 (0.11-1.37)	0.143
Non-autoimmune thyroiditis (n = 27,975)										
Hypothyroidism	658/5,516 (11.9)	776/22,459 (3.5)	3.84 (3.45-4.29)	<0.001*	3.91 (3.49-4.37)	<0.001*	3.43 (3.06-3.85)	<0.001*	3.88 (3.47-4.35)	<0.001*
Hyperthyroidism	352/5,516 (6.4)	608/22,459 (2.7)	2.46 (2.15-2.81)	<0.001*	2.48 (2.16-2.86)	<0.001*	1.92 (1.66-2.23)	<0.001*	N/A	
Thyroiditis	189/5,516 (3.4)	240/22,459 (1.1)	3.31 (2.73-4.01)	<0.001*	3.30 (2.70-4.03)	<0.001*	2.39 (1.94-2.95)	<0.001*	N/A	
Autoimmune thyroiditis	0/5,516 (0.0)	0/22,459 (0.0)	N/A		N/A		N/A		N/A	
Graves' disease	40/5,516 (0.7)	70/22,459 (0.3)	2.34 (1.59-3.46)	<0.001*	2.43 (1.63-3.63)	<0.001*	N/A		2.06 (1.36-3.13)	0.001*
Autoimmune thyroiditis (n = 355)										
Hypothyroidism	52/150 (34.7)	107/205 (52.2)	0.47 (0.31-0.74)	0.001*	0.49 (0.31-0.79)	0.003*	0.52 (0.32-0.84)	0.008*	0.48 (0.30-0.78)	0.003*
Hyperthyroidism	17/150 (11.3)	54/205 (26.3)	0.35 (0.19-0.63)	0.001*	0.32 (0.17-0.60)	0.001*	0.33 (0.17-0.64)	0.001*	N/A	
Thyroiditis	150/150 (100.0)	205/205 (100.0)	N/A		N/A		N/A		N/A	
Autoimmune thyroiditis	150/150 (100.0)	205/205 (100.0)	N/A		N/A		N/A		N/A	
Graves' disease	1/150 (0.7)	7/205 (3.4)	0.18 (0.02-1.51)	0.115	0.05 (0.00-0.54)	0.014*	N/A		0.04 (0.00-0.51)	0.013*

* Un-conditional logistic regression, Significance at $P < 0.05$. † Model 1 was adjusted for age, sex, income, region of residence, total cholesterol, systolic blood pressure, diastolic blood pressure, fasting blood glucose, obesity, smoking, alcohol consumption, and Charlson comorbidity index scores. ‡ Model 2 was adjusted for model 1 plus hypothyroidism, hyperthyroidism, and thyroiditis. § Model 3 was adjusted for model 1 plus hypothyroidism, autoimmune thyroiditis, and Graves' disease.

Table S3. Subgroup analyses according to obesity, alcohol consumption, and smoking in each thyroid disease calculating odds ratios (95% confidence intervals) for thyroid cancer in Study I.

Characteristics	Thyroid cancer	Control	Odds ratios for thyroid cancer							
	(exposure/total, %)	(exposure/total, %)	Crude	*P*-value	Model 1†	*P*-value	Model 2‡	*P*-value	Model 3§	*P*-value

Underweight (n = 593)										
Hypothyroidism	10/76 (13.2)	15/517 (2.9)	5.17 (2.20-12.17)	<0.001*	4.52 (1.86-11.00)	0.001*	4.33 (1.72-10.88)	0.002*	4.41 (1.81-10.77)	0.001*
Hyperthyroidism	5/76 (6.6)	18/517 (3.5)	1.81 (0.64-5.10)	0.260	1.83 (0.64-5.24)	0.263	1.20 (0.38-3.75)	0.754	N/A	
Thyroiditis	2/76 (2.6)	8/517 (1.6)	1.58 (0.32-7.75)	0.570	1.50 (0.29-7.74)	0.630	1.42 (0.27-7.37)	0.680	N/A	
Autoimmune thyroiditis	1/76 (1.3)	3/517 (0.6)	2.40 (0.24-23.68)	0.453	2.65 (0.26-26.66)	0.407	N/A		1.90 (0.17-20.80)	0.600
Graves' disease	0/76 (0.0)	2/517 (0.4)	N/A		N/A		N/A		N/A	
Normal weight (n = 10,260)										
Hypothyroidism	253/1,872 (13.5)	350/8,388 (4.2)	3.54 (2.98-4.20)	<0.001*	3.69 (3.10-4.40)	<0.001*	2.98 (2.48-3.59)	<0.001*	3.46 (2.88-4.15)	<0.001*
Hyperthyroidism	137/1,872 (7.3)	261/8,388 (3.1)	2.43 (1.97-3.01)	<0.001*	2.44 (1.96-3.03)	<0.001*	1.90 (1.51-2.40)	<0.001*	N/A	
Thyroiditis	136/1,872 (7.3)	177/8,388 (2.1)	3.58 (2.84-4.50)	<0.001*	3.52 (2.78-4.47)	<0.001*	2.21 (1.72-2.86)	<0.001*	N/A	
Autoimmune thyroiditis	55/1,872 (2.9)	74/8,388 (0.9)	3.33 (2.34-4.74)	<0.001*	3.29 (2.28-4.73)	<0.001*	N/A		1.78 (1.21-2.63)	0.003*
Graves' disease	15/1,872 (0.8)	38/8,388 (0.5)	1.77 (0.97-3.22)	0.063	1.64 (0.88-3.06)	0.118	N/A		1.36 (0.70-2.62)	0.362
Overweight (n = 7,818)										
Hypothyroidism	194/1,611 (12.0)	237/6,207 (3.8)	3.53 (2.89-4.31)	<0.001*	3.55 (2.88-4.36)	<0.001*	2.99 (2.41-3.71)	<0.001*	3.42 (2.77-4.22)	<0.001*
Hyperthyroidism	117/1,611 (7.3)	186/6,207 (3.0)	2.55 (2.01-3.23)	<0.001*	2.59 (2.02-3.31)	<0.001*	1.91 (1.48-2.48)	<0.001*	N/A	
Thyroiditis	85/1,611 (5.3)	141/6,207 (2.3)	2.43 (1.84-3.20)	<0.001*	2.41 (1.81-3.21)	<0.001*	1.59 (1.17-2.15)	0.003*	N/A	
Autoimmune thyroiditis	37/1,611 (2.3)	69/6,207 (1.1)	2.12 (1.41-3.17)	<0.001*	2.21 (1.46-3.35)	<0.001*	N/A		1.38 (0.89-2.13)	0.153
Graves' disease	12/1,611 (0.7)	18/6,207 (0.3)	2.61 (1.25-5.42)	0.010*	2.73 (1.28-5.83)	0.009*	N/A		2.39 (1.10-5.21)	0.028*
Obese (n = 9,659)										

Hypothyroidism	253/2,107 (12.0)	281/7,552 (3.7)	3.64 (3.04-4.35)	<0.001*	3.71 (3.09-4.46)	<0.001*	3.17 (2.62-3.84)	<0.001*	3.45 (2.86-4.16)	<0.001*
Hyperthyroidism	110/2,107 (5.2)	197/7,552 (2.6)	2.08 (1.64-2.64)	<0.001*	2.05 (1.60-2.62)	<0.001*	1.46 (1.13-1.90)	0.004*	N/A	
Thyroiditis	116/2,107 (5.5)	119/7,552 (1.6)	3.71 (2.86-4.82)	<0.001*	3.66 (2.79-4.79)	<0.001*	2.62 (1.98-3.49)	<0.001*	N/A	
Autoimmune thyroiditis	57/2,107 (2.7)	59/7,552 (0.8)	3.57 (2.47-5.16)	<0.001*	3.43 (2.34-5.02)	<0.001*	N/A		2.25 (1.51-3.36)	<0.001*
Graves' disease	14/2,107 (0.7)	19/7,552 (0.3)	2.68 (1.34-5.36)	0.005*	2.72 (1.33-5.55)	0.006*	N/A		2.00 (0.95-4.25)	0.070
Non-smoker (n = 24,376)										
Hypothyroidism	664/4,959 (13.4)	843/19,417 (4.3)	3.46 (3.11-3.86)	<0.001*	3.55 (3.18-3.96)	<0.001*	2.96 (2.63-3.32)	<0.001*	3.33 (2.97-3.72)	<0.001*
Hyperthyroidism	328/4,959 (6.6)	600/19,417 (3.1)	2.24 (1.95-2.57)	<0.001*	2.27 (1.97-2.62)	<0.001*	1.68 (1.44-1.95)	<0.001*	N/A	
Thyroiditis	326/4,959 (6.6)	428/19,417 (2.2)	3.17 (2.73-3.67)	<0.001*	3.16 (2.71-3.68)	<0.001*	2.11 (1.80-2.48)	<0.001*	N/A	
Autoimmune thyroiditis	148/4,959 (3.0)	197/19,417 (1.0)	3.04 (2.45-3.77)	<0.001*	3.03 (2.43-3.79)	<0.001*	N/A		1.84 (1.45-2.32)	<0.001*
Graves' disease	38/4,959 (0.8)	71/19,417 (0.4)	2.12 (1.43-3.15)	<0.001*	2.17 (1.44-3.26)	<0.001*	N/A		1.78 (1.17-2.73)	0.008*
Past and current smoker (n = 3,954)										
Hypothyroidism	46/707 (6.5)	40/3,247 (1.2)	6.14 (3.96-9.52)	<0.001*	6.99 (4.39-11.14)	<0.001*	6.19 (3.85-9.96)	<0.001*	6.96 (4.37-11.10)	<0.001*
Hyperthyroidism	41/707 (5.8)	62/3,247 (1.9)	3.23 (2.16-4.84)	<0.001*	3.21 (2.09-4.91)	<0.001*	2.51 (1.60-3.93)	<0.001*	N/A	
Thyroiditis	13/707 (1.8)	17/3,247 (0.5)	3.79 (1.83-7.87)	<0.001*	4.27 (2.02-9.03)	<0.001*	3.29 (1.52-7.13)	0.003	N/A	
Autoimmune thyroiditis	2/707 (0.3)	8/3,247 (0.3)	1.20 (0.26-5.69)	0.815	1.37 (0.29-6.55)	0.694	N/A		1.17 (0.23-5.87)	0.852
Graves' disease	3/707 (0.4)	6/3,247 (0.2)	2.34 (0.58-9.41)	0.230	1.79 (0.41-7.87)	0.443	N/A		1.24 (0.24-6.26)	0.799
Alcohol < 1 time a week (n = 22,060)										
Hypothyroidism	610/4,430 (13.8)	746/17,630 (4.2)	3.68 (3.29-4.12)	<0.001*	3.75 (3.33-4.21)	<0.001*	3.10 (2.75-3.51)	<0.001*	3.52 (3.13-3.97)	<0.001*

Hyperthyroidism	310/4,430 (7.0)	540/17,630 (3.1)	2.39 (2.07-2.76)	<0.001*	2.43 (2.10-2.82)	<0.001*	1.80 (1.54-2.11)	<0.001*	N/A	
Thyroiditis	293/4,430 (6.6)	377/17,630 (2.1)	3.29 (2.81-3.85)	<0.001*	3.31 (2.81-3.89)	<0.001*	2.21 (1.86-2.62)	<0.001*	N/A	
Autoimmune thyroiditis	127/4,430 (2.9)	171/17,630 (1.0)	3.05 (2.42-3.85)	<0.001*	3.08 (2.42-3.92)	<0.001*	N/A		1.84 (1.43-2.38)	<0.001*
Graves' disease	35/4,430 (0.8)	66/17,630 (0.4)	2.13 (1.41-3.21)	<0.001*	2.16 (1.42-3.31)	<0.001*	N/A		1.87 (1.20-2.92)	0.006*
Alcohol ≥ 1 time a week (n = 6,270)										
Hypothyroidism	100/1,236 (8.1)	137/5,034 (2.7)	3.09 (2.36-4.04)	<0.001*	3.18 (2.41-4.20)	<0.001*	2.76 (2.06-3.69)	<0.001*	3.04 (2.29-4.04)	<0.001*
Hyperthyroidism	59/1,236 (4.8)	122/5,034 (2.4)	1.98 (1.44-2.72)	<0.001*	1.94 (1.40-2.70)	<0.001*	1.49 (1.05-2.10)	0.025*	N/A	
Thyroiditis	46/1,236 (3.7)	68/5,034 (1.4)	2.74 (1.87-4.01)	<0.001*	2.54 (1.71-3.78)	<0.001*	1.70 (1.12-2.59)	0.013*	N/A	
Autoimmune thyroiditis	23/1,236 (1.9)	34/5,034 (0.7)	2.67 (1.56-4.56)	<0.001*	2.41 (1.38-4.19)	0.002*	N/A		1.56 (0.87-2.77)	0.133
Graves' disease	6/1,236 (0.5)	11/5,034 (0.2)	2.20 (0.81-5.97)	0.121	1.92 (0.66-5.53)	0.229	N/A		1.20 (0.41-3.54)	0.738

* Un-conditional logistic regression, Significance at $P < 0.05$. † Model 1 was adjusted for age, sex, income, region of residence, total cholesterol, systolic blood pressure, diastolic blood pressure, fasting blood glucose, obesity, smoking, alcohol consumption, and Charlson comorbidity index scores. ‡ Model 2 was adjusted for model 1 plus hypothyroidism, hyperthyroidism, and thyroiditis. § Model 3 was adjusted for model 1 plus hypothyroidism, autoimmune thyroiditis, and Graves' disease.

Table S4. Subgroup analyses according to CCI, total cholesterol, blood pressure, and fasting blood glucose in each thyroid disease calculating odds ratios (95% confidence intervals) for thyroid cancer in Study I.

Characteristics	Thyroid cancer	Control	Odds ratios for thyroid cancer							
	(exposure/total, %)	(exposure/total, %)	Crude	*P*-value	Model 1†	*P*-value	Model 2‡	*P*-value	Model 3§	*P*-value
CCI 0 score (n = 21,098)										
Hypothyroidism	447/3,441 (13.0)	689/17,657 (3.9)	3.70 (3.26-4.20)	<0.001*	3.70 (3.26-4.20)	<0.001*	3.05 (2.67-3.49)	<0.001*	3.49 (3.07-3.98)	<0.001*
Hyperthyroidism	221/3,441 (6.4)	506/17,657 (2.9)	2.32 (1.97-2.73)	<0.001*	2.32 (1.97-2.73)	<0.001*	1.69 (1.42-2.00)	<0.001*	N/A	
Thyroiditis	213/3,441 (6.2)	341/17,657 (1.9)	3.34 (2.80-3.99)	<0.001*	3.35 (2.81-4.00)	<0.001*	2.16 (1.78-2.61)	<0.001*	N/A	

Autoimmune thyroiditis	94/3,441 (2.7)	162/17,657 (0.9)	3.02 (2.33-3.90)	<0.001*	2.99 (2.31-3.87)	<0.001*	N/A		1.69 (1.28-2.23)	<0.001*
Graves' disease	24/3,441 (0.7)	62/17,657 (0.4)	1.98 (1.23-3.17)	0.005*	1.98 (1.23-3.18)	0.005*	N/A		1.69 (1.03-2.76)	0.037*
CCI 1 score (n = 3,668)										
Hypothyroidism	110/918 (12.0)	122/2,750 (4.4)	2.90 (2.21-3.80)	<0.001*	2.84 (2.16-3.73)	<0.001*	2.47 (1.86-3.26)	<0.001*	2.66 (2.02-3.51)	<0.001*
Hyperthyroidism	59/918 (6.4)	93/2,750 (3.4)	1.95 (1.39-2.73)	<0.001*	1.96 (1.40-2.75)	<0.001*	1.57 (1.10-2.23)	0.013*	N/A	
Thyroiditis	56/918 (6.1)	60/2,750 (2.2)	2.86 (1.96-4.16)	<0.001*	2.88 (1.98-4.21)	<0.001*	2.28 (1.54-3.36)	<0.001*	N/A	
Autoimmune thyroiditis	25/918 (2.7)	26/2,750 (1.0)	2.84 (1.63-4.95)	<0.001*	2.82 (1.61-4.93)	<0.001*	N/A		2.15 (1.20-3.83)	0.010*
Graves' disease	10/918 (1.1)	06/2,750 (0.2)	5.05 (1.83-13.95)	0.002*	4.69 (1.69-13.00)	0.003*	N/A		4.18 (1.48-11.85)	0.007*
CCI ≥ 2 score (n = 3,564)										
Hypothyroidism	153/1,307 (11.7)	72/2,257 (3.2)	4.04 (3.01-5.42)	<0.001*	4.73 (3.44-6.49)	<0.001*	4.04 (2.92-5.59)	<0.001*	1.81 (1.16-2.82)	0.009*
Hyperthyroidism	89/1,307 (6.8)	63/2,257 (2.8)	2.51 (1.80-3.51)	<0.001*	2.93 (2.04-4.21)	<0.001*	2.27 (1.56-3.32)	<0.001*	N/A	
Thyroiditis	70/1,307 (5.4)	44/2,257 (2.0)	2.78 (1.88-4.10)	<0.001*	2.68 (1.74-4.13)	<0.001*	1.81 (1.16-2.82)	0.009*	N/A	
Autoimmune thyroiditis	31/1,307 (2.4)	17/2,257 (0.8)	3.11 (1.71-5.68)	<0.001*	2.91 (1.50-5.66)	0.002*	N/A		2.08 (1.06-4.05)	0.032*
Graves' disease	7/1,307 (0.5)	9/2,257 (0.4)	1.28 (0.47-3.48)	0.635	1.05 (0.34-3.22)	0.933	N/A		0.57 (0.18-1.80)	0.337
Total cholesterol < 200 mg/dL (n = 14,427)										
Hypothyroidism	388/3,021 (12.8)	462/11,406 (4.1)	3.55 (3.08-4.09)	<0.001*	3.58 (3.09-4.14)	<0.001*	3.00 (2.58-3.50)	<0.001*	3.37 (2.90-3.92)	<0.001*
Hyperthyroidism	210/3,021 (7.0)	352/11,406 (3.1)	2.35 (1.97-2.80)	<0.001*	2.42 (2.02-2.90)	<0.001*	1.84 (1.52-2.22)	<0.001*	N/A	
Thyroiditis	179/3,021 (5.9)	233/11,406 (2.0)	3.06 (2.50-3.73)	<0.001*	3.01 (2.45-3.70)	<0.001*	2.06 (1.65-2.56)	<0.001*	N/A	
Autoimmune thyroiditis	81/3,021 (2.7)	106/11,406 (0.9)	2.96 (2.21-3.97)	<0.001*	2.95 (2.18-3.99)	<0.001*	N/A		1.80 (1.31-2.47)	<0.001*

Graves' disease	22/3,021 (0.7)	45/11,406 (0.4)	1.86 (1.11-3.10)	0.018*	1.98 (1.17-3.34)	0.011*	N/A		1.59 (0.92-2.74)	0.100
Total cholesterol ≥ 200 to < 240 mg/dL (n = 9,774)										
Hypothyroidism	228/1,882 (12.1)	291/7,892 (3.7)	3.61 (3.01-4.34)	<0.001*	3.82 (3.17-4.61)	<0.001*	3.14 (2.58-3.83)	<0.001*	3.55 (2.92-4.30)	<0.001*
Hyperthyroidism	115/1,882 (6.1)	204/7,892 (2.6)	2.44 (1.93-3.09)	<0.001*	2.35 (1.84-3.00)	<0.001*	1.67 (1.29-2.17)	<0.001*	N/A	
Thyroiditis	117/1,882 (6.2)	154/7,892 (2.0)	3.33 (2.60-4.26)	<0.001*	3.41 (2.64-4.40)	<0.001*	2.28 (1.74-3.00)	<0.001*	N/A	
Autoimmune thyroiditis	55/1,882 (2.9)	72/7,892 (0.9)	3.25 (2.28-4.64)	<0.001*	3.19 (2.20-4.62)	<0.001*	N/A		1.88 (1.27-2.79)	0.002*
Graves' disease	15/1,882 (0.8)	21/7,892 (0.3)	3.00 (1.54-5.83)	0.001*	2.96 (1.48-5.89)	0.002*	N/A		2.29 (1.10-4.76)	0.027*
Total cholesterol ≥ 240 mg/dL (n = 4,129)										
Hypothyroidism	94/763 (12.3)	130/3,366 (3.9)	3.55 (2.68-4.70)	<0.001*	3.61 (2.70-4.82)	<0.001*	3.05 (2.25-4.13)	<0.001*	3.51 (2.61-4.70)	<0.001*
Hyperthyroidism	44/763 (5.8)	106/3,366 (3.2)	1.89 (1.32-2.72)	0.001*	1.95 (1.35-2.83)	<0.001*	1.48 (1.01-2.19)	0.047*	N/A	
Thyroiditis	43/763 (5.6)	58/3,366 (1.7)	3.40 (2.27-5.10)	<0.001*	3.37 (2.22-5.12)	<0.001*	2.05 (1.31-3.22)	0.002*	N/A	
Autoimmune thyroiditis	14/763 (1.8)	27/3,366 (0.8)	2.30 (1.20-4.41)	0.012*	2.45 (1.26-4.79)	0.009*	N/A		1.51 (0.75-3.04)	0.245
Graves' disease	4/763 (0.5)	11/3,366 (0.3)	1.61 (0.51-5.08)	0.415	1.20 (0.35-4.18)	0.771	N/A		1.30 (0.37-4.54)	0.680
SBP < 140 mmHg and DBP < 90 mmHg (n = 22,777)										
Hypothyroidism	573/4,528 (12.7)	766/18,249 (4.2)	3.35 (2.99-3.76)	<0.001*	3.40 (3.02-3.83)	<0.001*	2.82 (2.49-3.19)	<0.001*	3.18 (2.82-3.59)	<0.001*
Hyperthyroidism	312/4,528 (6.9)	549/18,249 (3.0)	2.39 (2.07-2.76)	<0.001*	2.39 (2.06-2.77)	<0.001*	1.80 (1.54-2.10)	<0.001*	N/A	
Thyroiditis	288/4,528 (6.4)	389/18,249 (2.1)	3.14 (2.69-3.67)	<0.001*	3.11 (2.64-3.66)	<0.001*	2.11 (1.78-2.51)	<0.001*	N/A	
Autoimmune thyroiditis	129/4,528 (2.9)	176/18,249 (1.0)	3.02 (2.40-3.80)	<0.001*	3.03 (2.39-3.85)	<0.001*	N/A		1.89 (1.47-2.43)	<0.001*
Graves' disease	37/4,528 (0.8)	68/18,249 (0.4)	2.21 (1.48-3.30)	<0.001*	2.11 (1.39-3.20)	0.001*	N/A		1.75 (1.13-2.70)	0.013*

SBP ≥ 140 mmHg or DBP ≥ 90 mmHg (n = 5,553)										
Hypothyroidism	137/1,138 (12.0)	117/4,415 (2.7)	5.11 (3.94-6.61)	<0.001*	5.30 (4.06-6.90)	<0.001*	4.57 (3.47-6.01)	<0.001*	5.14 (3.93-6.73)	<0.001*
Hyperthyroidism	57/1,138 (5.0)	113/4,415 (2.6)	1.99 (1.44-2.76)	<0.001*	2.02 (1.45-2.82)	<0.001*	1.42 (0.99-2.03)	0.057	N/A	
Thyroiditis	51/1,138 (4.5)	56/4,415 (1.3)	3.67 (2.50-5.41)	<0.001*	3.70 (2.49-5.49)	<0.001*	2.27 (1.49-3.47)	<0.001*	N/A	
Autoimmune thyroiditis	21/1,138 (1.9)	29/4,415 (0.7)	2.86 (1.62-5.04)	<0.001*	2.69 (1.50-4.82)	0.001*	N/A		1.38 (0.74-2.57)	0.313
Graves' disease	4/1,138 (0.4)	9/4,415 (0.2)	1.70 (0.52-5.55)	0.376	1.98 (0.61-6.51)	0.258	N/A		1.41 (0.40-4.98)	0.592
Fasting blood glucose < 100 mg/dL (n = 19,526)										
Hypothyroidism	508/3,915 (13.0)	617/15,611 (4.0)	3.69 (3.26-4.17)	<0.001*	3.74 (3.29-4.25)	<0.001*	3.13 (2.74-3.58)	<0.001*	3.52 (3.09-4.01)	<0.001*
Hyperthyroidism	258/3,915 (6.6)	462/15,611 (3.0)	2.32 (1.98-2.71)	<0.001*	2.31 (1.97-2.72)	<0.001*	1.74 (1.47-2.06)	<0.001*	N/A	
Thyroiditis	243/3,915 (6.2)	331/15,611 (2.1)	3.08 (2.60-3.66)	<0.001*	3.06 (2.56-3.65)	<0.001*	2.01 (1.67-2.43)	<0.001*	N/A	
Autoimmune thyroiditis	108/3,915 (2.8)	152/15,611 (1.0)	2.91 (2.26-3.73)	<0.001*	2.92 (2.26-3.78)	<0.001*	N/A		1.71 (1.30-2.24)	<0.001*
Graves' disease	30/3,915 (0.8)	56/15,611 (0.4)	2.15 (1.38-3.36)	0.001*	2.15 (1.36-3.41)	0.001*	N/A		1.79 (1.11-2.90)	0.018*
Fasting blood glucose ≥ 100 mg/dL (n = 8,804)										
Hypothyroidism	202/1,751 (11.5)	266/7,053 (3.8)	3.35 (2.76-4.06)	<0.001*	3.44 (2.82-4.19)	<0.001*	2.87 (2.33-3.53)	<0.001*	2.40 (1.77-3.24)	<0.001*
Hyperthyroidism	111/1,751 (6.3)	200/7,053 (2.8)	2.31 (1.82-2.94)	<0.001*	2.29 (1.79-2.93)	<0.001*	1.68 (1.29-2.18)	<0.001*	N/A	
Thyroiditis	96/1,751 (5.5)	114/7,053 (1.6)	3.53 (2.67-4.66)	<0.001*	3.45 (2.59-4.60)	<0.001*	2.40 (1.77-3.24)	<0.001*	N/A	
Autoimmune thyroiditis	42/1,751 (2.4)	53/7,053 (0.8)	3.24 (2.15-4.88)	<0.001*	3.09 (2.03-4.73)	<0.001*	N/A		2.04 (1.31-3.16)	0.002*
Graves' disease	11/1,751 (0.6)	21/7,053 (0.3)	2.10 (1.01-4.36)	0.048*	2.01 (0.94-4.27)	0.071	N/A		1.55 (0.70-3.44)	0.281

* Un-conditional logistic regression, Significance at *P* < 0.05. † Model 1 was adjusted for age, sex, income, region of residence, total cholesterol, systolic blood pressure, diastolic blood pressure, fasting blood glucose, obesity, smoking, alcohol consumption, and Charlson comorbidity index scores. ‡ Model 2 was adjusted for model 1 plus hypothyroidism, hyperthyroidism, and thyroiditis. § Model 3 was adjusted for model 1 plus hypothyroidism, autoimmune thyroiditis, and Graves' disease.

Table S5. Subgroup analyses according to age, sex, income, and region of residence in each thyroid disease calculating odds ratios (95% confidence intervals) for thyroid cancer in Study II.

Characteristics	Thyroid cancer	Control	Odds ratios for thyroid cancer							
	(exposure/total, %)	(exposure/total, %)	Crude	*P*-value	Model 1†	*P*-value	Model 2‡	*P*-value	Model 3§	*P*-value
Age ≤ 60 years old (n = 294,232)										
Hypothyroidism	16,900/147,116 (11.5)	5,760/147,116 (3.9)	3.19 (3.09-3.29)	<0.001*	1.35 (1.31-1.40)	<0.001*	1.34 (1.29-1.39)	<0.001*	1.36 (1.31-1.40)	<0.001*
Hyperthyroidism	9,926/147,116 (6.8)	4,353/147,116 (3.0)	2.37 (2.29-2.46)	<0.001*	0.84 (0.80-0.87)	<0.001*	0.79 (0.75-0.82)	<0.001*	N/A	
Thyroiditis	9,962/147,116 (6.8)	3,161/147,116 (2.2)	3.31 (3.18-3.45)	<0.001*	1.43 (1.36-1.49)	<0.001*	1.40 (1.33-1.46)	<0.001*	N/A	
Autoimmune thyroiditis	4,585/147,116 (3.1)	1,369/147,116 (0.9)	3.43 (3.22-3.64)	<0.001*	1.23 (1.15-1.32)	<0.001*	N/A		1.17 (1.10-1.26)	<0.001*
Graves' disease	3,748/147,116 (2.6)	1,548/147,116 (1.1)	2.46 (2.32-2.61)	<0.001*	0.72 (0.66-0.77)	<0.001*	N/A		0.68 (0.63-0.74)	<0.001*
Age > 60 years old (n = 132,332)										
Hypothyroidism	8,566/66,166 (13.0)	3,234/66,166 (4.9)	2.89 (2.78-3.02)	<0.001*	1.19 (1.14-1.25)	<0.001*	1.19 (1.13-1.25)	<0.001*	1.19 (1.14-1.25)	<0.001*
Hyperthyroidism	4,781/66,166 (7.2)	2,129/66,166 (3.2)	2.34 (2.22-2.47)	<0.001*	0.85 (0.80-0.91)	<0.001*	0.83 (0.78-0.88)	<0.001*	N/A	
Thyroiditis	4,179/66,166 (6.3)	1,486/66,166 (2.3)	2.93 (2.76-3.12)	<0.001*	1.31 (1.22-1.40)	<0.001*	1.29 (1.20-1.38)	<0.001*	N/A	
Autoimmune thyroiditis	1,977/66,166 (3.0)	653/66,166 (1.0)	3.09 (2.83-3.38)	<0.001*	1.19 (1.08-1.32)	0.001*	N/A		1.17 (1.05-1.29)	0.003*
Graves' disease	1,556/66,166 (2.4)	659/66,166 (1.0)	2.39 (2.18-2.62)	<0.001*	0.73 (0.65-0.81)	<0.001*	N/A		0.71 (0.64-0.80)	<0.001*
Males (n = 75,054)										

Hypothyroidism	1,698/37,527 (4.5)	298/37,527 (0.8)	5.92 (5.23-6.70)	<0.001*	2.27 (1.97-2.61)	<0.001*	2.22 (1.93-2.56)	<0.001*	2.24 (1.95-2.58)	<0.001*
Hyperthyroidism	1,510/37,527 (4.0)	433/37,527 (1.2)	3.59 (3.22-4.00)	<0.001*	0.92 (0.80-1.06)	0.234	0.82 (0.71-0.94)	0.005*	N/A	
Thyroiditis	1,017/37,527 (2.7)	137/37,527 (0.4)	7.59 (6.35-9.07)	<0.001*	2.94 (2.41-3.58)	<0.001*	2.87 (2.35-3.50)	<0.001*	N/A	
Autoimmune thyroiditis	436/37,527 (1.2)	50/37,527 (0.1)	8.81 (6.57-11.81)	<0.001*	2.61 (1.88-3.62)	<0.001*	N/A		2.42 (1.74-3.37)	<0.001*
Graves' disease	634/37,527 (1.7)	155/37,527 (0.4)	4.14 (3.47-4.94)	<0.001*	0.81 (0.64-1.02)	0.077	N/A		0.75 (0.59-0.95)	0.016*
Females (n = 351,510)										
Hypothyroidism	23,768/175,755 (13.5)	8,696/175,755 (5.0)	3.00 (2.93-3.08)	<0.001*	1.29 (1.25-1.33)	<0.001*	1.27 (1.24-1.31)	<0.001*	1.29 (1.25-1.33)	<0.001*
Hyperthyroidism	13,197/175,755 (7.5)	6,049/175,755 (3.4)	2.28 (2.21-2.35)	<0.001*	0.84 (0.81-0.87)	<0.001*	0.80 (0.77-0.83)	<0.001*	N/A	
Thyroiditis	13,124/175,755 (7.5)	4,510/175,755 (2.6)	3.06 (2.96-3.17)	<0.001*	1.36 (1.31-1.41)	<0.001*	1.33 (1.28-1.39)	<0.001*	N/A	
Autoimmune thyroiditis	6,126/175,755 (3.5)	1,972/175,755 (1.1)	3.18 (3.02-3.35)	<0.001*	1.20 (1.14-1.28)	<0.001*	N/A		1.16 (1.10-1.23)	<0.001*
Graves' disease	4,670/175,755 (2.7)	2,052/175,755 (1.2)	2.31 (2.19-2.43)	<0.001*	0.72 (0.67-0.77)	<0.001*	N/A		0.69 (0.65-0.74)	<0.001*
Low income (n = 250,721)										
Hypothyroidism	14,285/116,439 (12.3)	5,173/134,282 (3.9)	3.49 (3.38-3.61)	<0.001*	1.39 (1.33-1.44)	<0.001*	1.36 (1.31-1.42)	<0.001*	1.38 (1.33-1.44)	<0.001*
Hyperthyroidism	8,380/116,439 (7.2)	3,864/134,282 (2.9)	2.62 (2.52-2.72)	<0.001*	0.85 (0.81-0.89)	<0.001*	0.80 (0.76-0.84)	<0.001*	N/A	
Thyroiditis	7,928/116,439 (6.8)	2,596/134,282 (1.9)	3.71 (3.54-3.88)	<0.001*	1.54 (1.46-1.62)	<0.001*	1.50 (1.43-1.58)	<0.001*	N/A	
Autoimmune thyroiditis	3,575/116,439 (3.1)	1,094/134,282 (0.8)	3.86 (3.60-4.13)	<0.001*	1.35 (1.24-1.45)	<0.001*	N/A		1.29 (1.19-1.40)	<0.001*
Graves' disease	3,049/116,439 (2.6)	1,357/134,282 (1.0)	2.63 (2.47-2.81)	<0.001*	0.68 (0.62-0.73)	<0.001*	N/A		0.64 (0.59-0.70)	<0.001*

High income (n = 175,843)										
Hypothyroidism	11,181/96,843 (11.6)	3,821/79,000 (4.8)	2.57 (2.47-2.67)	<0.001*	1.19 (1.14-1.24)	<0.001*	1.19 (1.14-1.24)	<0.001*	1.19 (1.14-1.25)	<0.001*
Hyperthyroidism	6,327/96,843 (6.5)	2,618/79,000 (3.3)	2.04 (1.95-2.14)	<0.001*	0.83 (0.78-0.87)	<0.001*	0.80 (0.76-0.85)	<0.001*	N/A	
Thyroiditis	6,213/96,843 (6.4)	2,051/79,000 (2.6)	2.57 (2.45-2.71)	<0.001*	1.21 (1.14-1.28)	<0.001*	1.20 (1.14-1.28)	<0.001*	N/A	
Autoimmune thyroiditis	2,987/96,843 (3.1)	928/79,000 (1.2)	2.68 (2.49-2.88)	<0.001*	1.09 (1.00-1.18)	0.056	N/A		1.06 (0.97-1.15)	0.202
Graves' disease	2,255/96,843 (2.3)	850/79,000 (1.1)	2.19 (2.02-2.37)	<0.001*	0.78 (0.71-0.86)	<0.001*	N/A		0.76 (0.69-0.84)	<0.001*
Urban (n = 197,934)										
Hypothyroidism	11,836/98,967 (12.0)	4,297/98,967 (4.3)	2.99 (2.89-3.10)	<0.001*	1.27 (1.22-1.33)	<0.001*	1.26 (1.20-1.31)	<0.001*	1.27 (1.22-1.33)	<0.001*
Hyperthyroidism	6,750/98,967 (6.8)	3,046/98,967 (3.1)	2.30 (2.21-2.41)	<0.001*	0.85 (0.80-0.89)	<0.001*	0.81 (0.77-0.85)	<0.001*	N/A	
Thyroiditis	7,054/98,967 (7.1)	2,336/98,967 (2.4)	3.18 (3.03-3.33)	<0.001*	1.41 (1.34-1.49)	<0.001*	1.39 (1.32-1.47)	<0.001*	N/A	
Autoimmune thyroiditis	3,379/98,967 (3.4)	1,082/98,967 (1.1)	3.20 (2.99-3.43)	<0.001*	1.22 (1.13-1.32)	<0.001*	N/A		1.19 (1.10-1.28)	<0.001*
Graves' disease	2,546/98,967 (2.6)	1,105/98,967 (1.1)	2.34 (2.18-2.51)	<0.001*	0.73 (0.67-0.80)	<0.001*	N/A		0.70 (0.64-0.77)	<0.001*
Rural (n = 228,630)										
Hypothyroidism	13,630/114,315 (11.9)	4,697/114,315 (4.1)	3.16 (3.05-3.27)	<0.001*	1.32 (1.27-1.38)	<0.001*	1.31 (1.26-1.37)	<0.001*	1.32 (1.27-1.38)	<0.001*
Hyperthyroidism	7,957/114,315 (7.0)	3,436/114,315 (3.0)	2.41 (2.32-2.51)	<0.001*	0.83 (0.79-0.88)	<0.001*	0.79 (0.75-0.83)	<0.001*	N/A	
Thyroiditis	7,087/114,315 (6.2)	2,311/114,315 (2.0)	3.20 (3.05-3.36)	<0.001*	1.37 (1.30-1.45)	<0.001*	1.34 (1.27-1.42)	<0.001*	N/A	
Autoimmune thyroiditis	3,183/114,315 (2.8)	940/114,315 (0.8)	3.45 (3.21-3.72)	<0.001*	1.22 (1.12-1.33)	<0.001*	N/A		1.17 (1.07-1.27)	<0.001*

Graves' disease	2,758/114,315 (2.4)	1,102/114,315 (1.0)	2.54 (2.37-2.72)	<0.001*	0.71 (0.65-0.77)	<0.001*	N/A		0.68 (0.63-0.75)	<0.001*

* Un-conditional logistic regression, Significance at $P < 0.05$. † Model 1 was adjusted for age, sex, income, region of residence, total cholesterol, systolic blood pressure, diastolic blood pressure, fasting blood glucose, obesity, smoking, alcohol consumption, Charlson comorbidity index scores, and the number of thyroid function tests. ‡ Model 2 was adjusted for model 1 plus hypothyroidism, hyperthyroidism, and thyroiditis. § Model 3 was adjusted for model 1 plus hypothyroidism, autoimmune thyroiditis, and Graves' disease.

Table S6. Subgroup analyses according to thyroid diseases in each thyroid disease calculating odds ratios (95% confidence intervals) for thyroid cancer in Study II.

Characteristics	Thyroid cancer	Control	Odds ratios for thyroid cancer							
	(exposure/total, %)	(exposure/total, %)	Crude	*P*-value	Model 1†	*P*-value	Model 2‡	*P*-value	Model 3§	*P*-value
Non-hypothyroidism (n = 392,104)										
Hypothyroidism	0/187,816 (0.0)	0//204,288 (0.0)	N/A		N/A		N/A		N/A	
Hyperthyroidism	10,915/187,816 (5.8)	4,857/204,288 (2.4)	2.53 (2.45-2.62)	<0.001*	0.85 (0.81-0.89)	<0.001*	0.82 (0.78-0.85)	<0.001*	N/A	
Thyroiditis	9,711/187,816 (5.2)	2,848/204,288 (1.4)	3.86 (3.70-4.02)	<0.001*	1.79 (1.71-1.88)	<0.001*	1.82 (1.73-1.91)	<0.001*	N/A	
Autoimmune thyroiditis	4,062/187,816 (2.2)	945/204,288 (0.5)	4.76 (4.43-5.11)	<0.001*	1.98 (1.83-2.15)	<0.001*	N/A		2.01 (1.85-2.17)	<0.001*
Graves' disease	3,913/187,816 (2.1)	1,594/204,288 (0.8)	2.70 (2.55-2.87)	<0.001*	0.75 (0.69-0.80)	<0.001*	N/A		0.73 (0.67-0.78)	<0.001*
Hypothyroidism (n= 34,460)										
Hypothyroidism	25,466/25,466 (100.0)	8,994/8,994 (100.0)	N/A		N/A		N/A		N/A	
Hyperthyroidism	3,792/25,466 (14.9)	1,625/8,994 (18.1)	0.79 (0.74-0.85)	<0.001*	0.69 (0.64-0.73)	<0.001*	0.70 (0.66-0.75)	<0.001*	N/A	
Thyroiditis	4,430/25,466 (17.4)	1,799/8,994 (20.0)	0.84 (0.79-0.90)	<0.001*	0.75 (0.71-0.80)	<0.001*	0.77 (0.72-0.82)	<0.001*	N/A	
Autoimmune thyroiditis	2,500/25,466 (9.8)	1,077/8,994 (12.0)	0.80 (0.74-0.86)	<0.001*	0.71 (0.65-0.76)	<0.001*	N/A		0.71 (0.66-0.77)	<0.001*
Graves' disease	1,391/25,466 (5.5)	613/8,994 (6.8)	0.79 (0.72-0.87)	<0.001*	0.65 (0.59-0.72)	<0.001*	N/A		0.66 (0.60-0.73)	<0.001*
Non-hyperthyroidism (n = 405,375)										

Hypothyroidism	21,674/198,575 (10.9)	7,369/206,800 (3.6)	3.32 (3.23-3.41)	<0.001*	1.30 (1.26-1.34)	<0.001*	1.27 (1.23-1.31)	<0.001*	1.29 (1.25-1.33)	<0.001*
Hyperthyroidism	0/198,575 (0.0)	0/206,800 (0.0)	N/A		N/A		N/A		N/A	
Thyroiditis	11,654/198,575 (5.9)	3,636/206,800 (1.8)	3.48 (3.36-3.62)	<0.001*	1.44 (1.38-1.51)	<0.001*	1.40 (1.34-1.46)	<0.001*	N/A	
Autoimmune thyroiditis	5,367/198,575 (2.7)	1,531/206,800 (0.7)	3.72 (3.52-3.94)	<0.001*	1.25 (1.17-1.33)	<0.001*	N/A		1.19 (1.11-1.27)	<0.001*
Graves' disease	871/198,575 (0.4)	395/206,800 (0.2)	2.30 (2.04-2.59)	<0.001*	1.35 (1.18-1.55)	<0.001*	N/A		1.33 (1.16-1.52)	<0.001*
Hyperthyroidism (n = 21,189)										
Hypothyroidism	3,792/14,707 (25.8)	1,625/6,482 (25.1)	1.04 (0.97-1.11)	0.274	0.94 (0.88-1.01)	0.097	0.94 (0.88-1.01)	0.100	0.95 (0.88-1.01)	0.116
Hyperthyroidism	14,707/14,707 (100.0)	6,482/6,482 (100.0)	N/A		N/A		N/A		N/A	
Thyroiditis	2,487/14,707 (16.9)	1,011/6,482 (15.6)	1.10 (1.02-1.19)	0.018*	0.99 (0.91-1.08)	0.841	1.00 (0.92-1.09)	0.944	N/A	
Autoimmune thyroiditis	1,195/14,707 (8.1)	491/6,482 (7.6)	1.08 (0.97-1.20)	0.173	0.97 (0.87-1.09)	0.621	N/A		0.99 (0.89-1.11)	0.880
Graves' disease	4,433/14,707 (30.1)	1,812/6,482 (28.0)	1.11 (1.04-1.19)	0.001*	0.93 (0.87-1.00)	0.042*	N/A		0.93 (0.87-1.00)	0.046*
Non-thyroiditis (n = 407,776)										
Hypothyroidism	21,036/199,141 (10.6)	7,195/208,635 (3.5)	3.31 (3.22-3.40)	<0.001*	1.44 (1.39-1.48)	<0.001*	1.45 (1.41-1.50)	<0.001*	1.45 (1.40-1.49)	<0.001*
Hyperthyroidism	12,220/199,141 (6.1)	5,471/208,635 (2.6)	2.43 (2.35-2.51)	<0.001*	0.85 (0.82-0.89)	<0.001*	0.82 (0.79-0.86)	<0.001*	N/A	
Thyroiditis	0/199,141 (0.0)	0/208,635 (0.0)	N/A		N/A		N/A		N/A	
Autoimmune thyroiditis	0/199,141 (0.0)	0/208,635 (0.0)	N/A		N/A		N/A		N/A	
Graves' disease	4,321/199,141 (2.2)	1,798/208,635 (0.9)	2.55 (2.41-2.70)	<0.001*	0.73 (0.69-0.79)	<0.001*	N/A		0.71 (0.66-0.76)	<0.001*
Thyroiditis (n = 18,788)										
Hypothyroidism	4,430/14,141 (31.3)	1,799/4,647 (38.7)	0.72 (0.67-0.77)	<0.001*	0.62 (0.58-0.67)	<0.001*	0.63 (0.59-0.68)	<0.001*	0.61 (0.57-0.66)	<0.001*

Hyperthyroidism	2,487/14,141 (17.6)	1,011/4,647 (21.8)	0.77 (0.71-0.83)	<0.001*	0.63 (0.58-0.69)	<0.001*	0.65 (0.60-0.71)	<0.001*	N/A	
Thyroiditis	14,141/14,141 (100.0)	4,647/4,647 (100.0)	N/A		N/A		N/A		N/A	
Autoimmune thyroiditis	6,562/14,141 (46.4)	2,022/4,647 (43.5)	1.12 (1.05-1.20)	0.001*	1.08 (1.01-1.16)	0.028*	N/A		1.16 (1.08-1.24)	<0.001*
Graves' disease	983/14,141 (7.0)	409/4,647 (8.8)	0.77 (0.69-0.87)	<0.001*	0.62 (0.55-0.71)	<0.001*	N/A		0.62 (0.55-0.71)	<0.001*
Non-autoimmune thyroiditis (n = 417,980)										
Hypothyroidism	22,966/206,720 (11.1)	7,917/211,260 (3.8)	3.21 (3.13-3.30)	<0.001*	1.39 (1.35-1.43)	<0.001*	1.39 (1.35-1.43)	<0.001*	1.40 (1.36-1.44)	<0.001*
Hyperthyroidism	13,512/206,720 (6.5)	5,991/211,260 (2.8)	2.40 (2.32-2.47)	<0.001*	0.84 (0.81-0.87)	<0.001*	0.80 (0.77-0.83)	<0.001*	N/A	
Thyroiditis	7,579/206,720 (3.7)	2,625/211,260 (1.2)	3.03 (2.89-3.16)	<0.001*	1.46 (1.39-1.54)	<0.001*	1.44 (1.37-1.51)	<0.001*	N/A	
Autoimmune thyroiditis	0/206,720 (0.0)	0/211,260 (0.0)	N/A		N/A		N/A		N/A	
Graves' disease	4,812/206,720 (2.3)	1,979/211,260 (0.9)	2.52 (2.39-2.66)	<0.001*	0.73 (0.69-0.78)	<0.001*	N/A		0.71 (0.66-0.76)	<0.001*
Autoimmune thyroiditis (n = 8,584)										
Hypothyroidism	2,500/6,562 (38.1)	1,077/2,022 (53.3)	0.54 (0.49-0.60)	<0.001*	0.50 (0.45-0.55)	<0.001*	0.50 (0.45-0.56)	<0.001*	0.50 (0.45-0.55)	<0.001*
Hyperthyroidism	1,195/6,562 (18.2)	491/2,022 (24.3)	0.69 (0.62-0.78)	<0.001*	0.64 (0.57-0.73)	<0.001*	0.66 (0.59-0.75)	<0.001*	N/A	
Thyroiditis	6,562/6,562 (100.0)	2,022/2,022 (100.0)	N/A		N/A		N/A		N/A	
Autoimmune thyroiditis	6,562/6,562 (100.0)	2,022/2,022 (100.0)	N/A		N/A		N/A		N/A	
Graves' disease	492/6,562 (7.5)	228/2,022 (11.3)	0.64 (0.54-0.75)	<0.001*	0.59 (0.49-0.69)	<0.001*	N/A		0.59 (0.50-0.70)	<0.001*
Non-Graves' disease (n = 419,053)										
Hypothyroidism	24,075/207,978 (11.6)	8,381/211,075 (4.0)	3.17 (3.09-3.25)	<0.001*	1.29 (1.25-1.33)	<0.001*	1.27 (1.23-1.30)	<0.001*	1.28 (1.24-1.32)	<0.001*
Hyperthyroidism	10,274/207,978 (4.9)	4,670/211,075 (2.2)	2.30 (2.22-2.38)	<0.001*	0.90 (0.86-0.94)	<0.001*	0.86 (0.83-0.90)	<0.001*	N/A	

Thyroiditis	13,158/207,978 (6.3)	4,238/211,075 (2.0)	3.30 (3.18-3.41)	<0.001*	1.40 (1.34-1.45)	<0.001*	1.36 (1.31-1.42)	<0.001*	N/A	
Autoimmune thyroiditis	6,070/207,978 (2.9)	1,794/211,075 (0.9)	3.51 (3.33-3.70)	<0.001*	1.24 (1.17-1.32)	<0.001*	N/A		1.18 (1.11-1.26)	<0.001*
Graves' disease	0/207,978 (0.0)	0/211,075 (0.0)	N/A		N/A		N/A		N/A	
Graves' disease (n = 7,511)										
Hypothyroidism	1,391/5,304 (26.2)	613/2,207 (27.8)	0.92 (0.83-1.03)	0.167	0.88 (0.78-0.98)	0.022*	0.88 (0.79-0.99)	0.032*	0.89 (0.79-1.00)	0.043*
Hyperthyroidism	4,433/5,304 (83.6)	1,812/2,207 (82.1)	1.11 (0.97-1.27)	0.120	0.90 (0.79-1.03)	0.128	0.91 (0.79-1.04)	0.166	N/A	
Thyroiditis	983/5,304 (18.5)	409/2,207 (18.5)	1.00 (0.88-1.14)	1.000	0.95 (0.83-1.09)	0.454	0.98 (0.86-1.12)	0.745	N/A	
Autoimmune thyroiditis	492/5,304 (9.3)	228/2,207 (10.3)	0.89 (0.75-1.05)	0.157	0.85 (0.72-1.01)	0.069	N/A		0.88 (0.74-1.05)	0.144
Graves' disease	5,304/5,304 (100.0)	2,207/2,207 (100.0)	N/A		N/A		N/A		N/A	

* Un-conditional logistic regression, Significance at $P < 0.05$. † Model 1 was adjusted for age, sex, income, region of residence, total cholesterol, systolic blood pressure, diastolic blood pressure, fasting blood glucose, obesity, smoking, alcohol consumption, Charlson comorbidity index scores, and the number of thyroid function tests. ‡ Model 2 was adjusted for model 1 plus hypothyroidism, hyperthyroidism, and thyroiditis. § Model 3 was adjusted for model 1 plus hypothyroidism, autoimmune thyroiditis, and Graves' disease.

Table S7. Subgroup analyses according to obesity, alcohol consumption, and smoking in each thyroid disease calculating odds ratios (95% confidence intervals) for thyroid cancer in Study II.

Characteristics	Thyroid cancer	Control	Odds ratios for thyroid cancer							
	(exposure/total, %)	(exposure/total, %)	Crude	*P*-value	Model 1†	*P*-value	Model 2‡	*P*-value	Model 3§	*P*-value
Underweight (n = 16,001)										
Hypothyroidism	866/6,637 (13.1)	378/9,364 (4.0)	3.57 (3.15-4.04)	<0.001*	1.42 (1.23-1.65)	<0.001*	1.37 (1.18-1.59)	<0.001*	1.40 (1.21-1.63)	<0.001*
Hyperthyroidism	610/6,637 (9.2)	351/9,364 (3.8)	2.60 (2.27-2.98)	<0.001*	0.89 (0.76-1.05)	0.182	0.84 (0.71-0.99)	0.042*	N/A	
Thyroiditis	505/6,637 (7.6)	199/9,364 (2.1)	3.79 (3.21-4.48)	<0.001*	1.58 (1.30-1.91)	<0.001*	1.52 (1.25-1.84)	<0.001*	N/A	
Autoimmune thyroiditis	243/6,637 (3.7)	90/9,364 (1.0)	3.92 (3.07-5.00)	<0.001*	1.34 (1.01-1.78)	0.041*	N/A		1.26 (0.95-1.67)	0.117

Graves' disease	225/6,637 (3.4)	109/9,364 (1.2)	2.98 (2.37-3.75)	<0.001*	0.86 (0.64-1.15)	0.298	N/A		0.81 (0.61-1.09)	0.163
Normal weight (n = 176,666)										
Hypothyroidism	10,721/84,051 (12.8)	3,999/92,615 (4.3)	3.24 (3.12-3.36)	<0.001*	1.32 (1.27-1.38)	<0.001*	1.31 (1.25-1.37)	<0.001*	1.34 (1.28-1.40)	<0.001*
Hyperthyroidism	6,509/84,051 (7.7)	3,047/92,615 (3.3)	2.47 (2.36-2.58)	<0.001*	0.84 (0.79-0.88)	<0.001*	0.80 (0.75-0.84)	<0.001*	N/A	
Thyroiditis	6,415/84,051 (7.6)	2,262/92,615 (2.4)	3.30 (3.14-3.47)	<0.001*	1.34 (1.27-1.42)	<0.001*	1.32 (1.24-1.39)	<0.001*	N/A	
Autoimmune thyroiditis	3,005/84,051 (3.6)	1,011/92,615 (1.1)	3.36 (3.13-3.61)	<0.001*	1.15 (1.06-1.25)	0.001*	N/A		1.12 (1.03-1.22)	0.010*
Graves' disease	2,423/84,051 (2.9)	1,076/92,615 (1.2)	2.53 (2.35-2.71)	<0.001*	0.70 (0.64-0.76)	<0.001*	N/A		0.67 (0.61-0.73)	<0.001*
Overweight (n = 97,311)										
Hypothyroidism	5,947/49,788 (11.9)	2,058/47,523 (4.3)	3.00 (2.85-3.16)	<0.001*	1.27 (1.20-1.35)	<0.001*	1.26 (1.19-1.34)	<0.001*	1.28 (1.20-1.36)	<0.001*
Hyperthyroidism	3,308/49,788 (6.6)	1,445/47,523 (3.0)	2.27 (2.13-2.42)	<0.001*	0.79 (0.73-0.85)	<0.001*	0.75 (0.70-0.81)	<0.001*	N/A	
Thyroiditis	3,172/49,788 (6.4)	1,041/47,523 (2.2)	3.04 (2.83-3.26)	<0.001*	1.34 (1.23-1.45)	<0.001*	1.32 (1.22-1.43)	<0.001*	N/A	
Autoimmune thyroiditis	1,528/49,788 (3.1)	444/47,523 (0.9)	3.36 (3.02-3.73)	<0.001*	1.24 (1.10-1.40)	0.000	N/A		1.22 (1.08-1.37)	0.002*
Graves' disease	1,163/49,788 (2.3)	472/47,523 (1.0)	2.38 (2.14-2.65)	<0.001*	0.72 (0.63-0.82)	<0.001*	N/A		0.69 (0.60-0.79)	<0.001*
Obese (n = 136,586)										
Hypothyroidism	7,932/72,806 (10.9)	2,559/63,780 (4.0)	2.93 (2.79-3.06)	<0.001*	1.27 (1.20-1.34)	<0.001*	1.25 (1.19-1.32)	<0.001*	1.28 (1.21-1.35)	<0.001*
Hyperthyroidism	4,280/72,806 (5.9)	1,639/63,780 (2.6)	2.37 (2.23-2.51)	<0.001*	0.88 (0.82-0.94)	<0.001*	0.84 (0.78-0.90)	<0.001*	N/A	
Thyroiditis	4,049/72,806 (5.6)	1,145/63,780 (1.8)	3.22 (3.01-3.44)	<0.001*	1.48 (1.37-1.59)	<0.001*	1.46 (1.35-1.57)	<0.001*	N/A	
Autoimmune thyroiditis	1,786/72,806 (2.5)	477/63,780 (0.8)	3.34 (3.02-3.69)	<0.001*	1.30 (1.16-1.46)	<0.001*	N/A		1.29 (1.15-1.44)	<0.001*
Graves' disease	1,493/72,806 (2.1)	550/63,780 (0.9)	2.40 (2.18-2.65)	<0.001*	0.73 (0.65-0.83)	<0.001*	N/A		0.70 (0.62-0.79)	<0.001*

Non-smoker (n = 356,022)										
Hypothyroidism	23,399/180,976 (12.9)	8,299/175,046 (4.7)	2.98 (2.91-3.06)	<0.001*	1.28 (1.25-1.32)	<0.001*	1.27 (1.23-1.31)	<0.001*	1.28 (1.24-1.32)	<0.001*
Hyperthyroidism	13,084/180,976 (7.2)	5,775/175,046 (3.3)	2.28 (2.21-2.36)	<0.001*	0.85 (0.82-0.88)	<0.001*	0.81 (0.78-0.84)	<0.001*	N/A	
Thyroiditis	12,990/180,976 (7.2)	4,352/175,046 (2.5)	3.03 (2.93-3.14)	<0.001*	1.36 (1.30-1.41)	<0.001*	1.33 (1.28-1.39)	<0.001*	N/A	
Autoimmune thyroiditis	6,072/180,976 (3.4)	1,893/175,046 (1.1)	3.18 (3.02-3.35)	<0.001*	1.21 (1.14-1.28)	<0.001*	N/A		1.17 (1.10-1.24)	<0.001*
Graves' disease	4,629/180,976 (2.6)	1,945/175,046 (1.1)	2.34 (2.21-2.46)	<0.001*	0.74 (0.69-0.79)	<0.001*	N/A		0.71 (0.67-0.76)	<0.001*
Past and current smoker (n = 70,542)										
Hypothyroidism	2,067/32,306 (6.4)	695/38,236 (1.8)	3.69 (3.38-4.03)	<0.001*	1.61 (1.45-1.78)	<0.001*	1.58 (1.43-1.76)	<0.001*	1.61 (1.46-1.79)	<0.001*
Hyperthyroidism	1,623/32,306 (5.0)	707/38,236 (1.9)	2.81 (2.57-3.07)	<0.001*	0.80 (0.71-0.90)	<0.001*	0.74 (0.66-0.83)	<0.001*	N/A	
Thyroiditis	1,151/32,306 (3.6)	295/38,236 (0.8)	4.75 (4.18-5.40)	<0.001*	1.99 (1.72-2.31)	<0.001*	1.96 (1.69-2.27)	<0.001*	N/A	
Autoimmune thyroiditis	490/32,306 (1.5)	129/38,236 (0.3)	4.55 (3.75-5.53)	<0.001*	1.50 (1.19-1.89)	0.001*	N/A		1.42 (1.12-1.78)	0.003*
Graves' disease	675/32,306 (2.1)	262/38,236 (0.7)	3.09 (2.68-3.57)	<0.001*	0.58 (0.47-0.70)	<0.001*	N/A		0.55 (0.45-0.67)	<0.001*
Alcohol < 1 time a week (n = 296,725)										
Hypothyroidism	19,750/150,844 (13.1)	6,950/145,881 (4.8)	3.01 (2.93-3.10)	<0.001*	1.27 (1.23-1.31)	<0.001*	1.26 (1.22-1.30)	<0.001*	1.27 (1.23-1.31)	<0.001*
Hyperthyroidism	11,264/150,844 (7.5)	4,884/145,881 (3.4)	2.33 (2.25-2.41)	<0.001*	0.84 (0.81-0.88)	<0.001*	0.81 (0.78-0.84)	<0.001*	N/A	
Thyroiditis	10,798/150,844 (7.2)	3,636/145,881 (2.5)	3.02 (2.90-3.13)	<0.001*	1.32 (1.26-1.38)	<0.001*	1.30 (1.24-1.35)	<0.001*	N/A	
Autoimmune thyroiditis	5,098/150,844 (3.4)	1,601/145,881 (1.1)	3.15 (2.98-3.34)	<0.001*	1.19 (1.11-1.27)	<0.001*	N/A		1.15 (1.08-1.22)	<0.001*
Graves' disease	3,953/150,844 (2.6)	1,648/145,881 (1.1)	2.35 (2.22-2.49)	<0.001*	0.72 (0.67-0.78)	<0.001*	N/A		0.70 (0.65-0.75)	<0.001*
Alcohol ≥ 1 time a week (n = 129,839)										

Hypothyroidism	5,716/62,438 (9.2)	2,044/67,401 (3.0)	3.22 (3.06-3.39)	<0.001*	1.42 (1.34-1.51)	<0.001*	1.39 (1.31-1.48)	<0.001*	1.42 (1.33-1.50)	<0.001*
Hyperthyroidism	3,443/62,438 (5.5)	1,598/67,401 (2.4)	2.40 (2.26-2.55)	<0.001*	0.84 (0.78-0.91)	<0.001*	0.78 (0.72-0.84)	<0.001*	N/A	
Thyroiditis	3,343/62,438 (5.4)	1,011/67,401 (1.5)	3.72 (3.46-3.99)	<0.001*	1.65 (1.52-1.79)	<0.001*	1.61 (1.49-1.75)	<0.001*	N/A	
Autoimmune thyroiditis	1,464/62,438 (2.3)	421/67,401 (0.6)	3.82 (3.43-4.26)	<0.001*	1.34 (1.18-1.52)	<0.001*	N/A		1.27 (1.12-1.44)	<0.001*
Graves' disease	1,351/62,438 (2.2)	559/67,401 (0.8)	2.64 (2.39-2.92)	<0.001*	0.71 (0.63-0.81)	<0.001*	N/A		0.68 (0.60-0.77)	<0.001*

* Un-conditional logistic regression, Significance at $P < 0.05$. † Model 1 was adjusted for age, sex, income, region of residence, total cholesterol, systolic blood pressure, diastolic blood pressure, fasting blood glucose, obesity, smoking, alcohol consumption, Charlson comorbidity index scores, and the number of thyroid function tests. ‡ Model 2 was adjusted for model 1 plus hypothyroidism, hyperthyroidism, and thyroiditis. § Model 3 was adjusted for model 1 plus hypothyroidism, autoimmune thyroiditis, and Graves' disease.

Table S8. Subgroup analyses according to CCI, total cholesterol, blood pressure, and fasting blood glucose in each thyroid disease calculating odds ratios (95% confidence intervals) for thyroid cancer in Study II.

Characteristics	Thyroid cancer	Control	Odds ratios for thyroid cancer							
	(exposure/total, %)	(exposure/total, %)	Crude	*P*-value	Model 1†	*P*-value	Model 2‡	*P*-value	Model 3§	*P*-value
CCI 0 score (n = 339,817)										
Hypothyroidism	19,723/168,375 (11.7)	6,912/171,442 (4.0)	3.16 (3.07-3.25)	<0.001*	1.28 (1.24-1.32)	<0.001*	1.27 (1.23-1.31)	<0.001*	1.28 (1.24-1.33)	<0.001*
Hyperthyroidism	11,332/168,375 (6.7)	5,125/171,442 (3.0)	2.34 (2.26-2.42)	<0.001*	0.79 (0.76-0.83)	<0.001*	0.75 (0.72-0.79)	<0.001*	N/A	
Thyroiditis	11,173/168,375 (6.6)	3,698/171,442 (2.2)	3.22 (3.10-3.35)	<0.001*	1.34 (1.29-1.40)	<0.001*	1.33 (1.27-1.39)	<0.001*	N/A	
Autoimmune thyroiditis	5,193/168,375 (3.1)	1,635/171,442 (1.0)	3.31 (3.13-3.50)	<0.001*	1.15 (1.08-1.23)	<0.001*	N/A		1.12 (1.05-1.19)	0.001*
Graves' disease	4,150/168,375 (2.5)	1,770/171,442 (1.0)	2.42 (2.29-2.56)	<0.001*	0.67 (0.62-0.72)	<0.001*	N/A		0.65 (0.60-0.70)	<0.001*
CCI 1 score (n = 58,055)										
Hypothyroidism	4,435/34,820 (12.7)	1,125/23,235 (4.8)	2.87 (2.68-3.07)	<0.001*	1.39 (1.29-1.50)	<0.001*	1.36 (1.26-1.47)	<0.001*	1.38 (1.28-1.48)	<0.001*
Hyperthyroidism	2,583/34,820 (7.4)	772/23,235 (3.3)	2.33 (2.15-2.53)	<0.001*	0.98 (0.89-1.07)	0.626	0.92 (0.84-1.02)	0.099	N/A	

Thyroiditis	2,354/34,820 (6.8)	521/23,235 (2.2)	3.16 (2.87-3.48)	<0.001*	1.56 (1.41-1.73)	<0.001*	1.51 (1.36-1.68)	<0.001*	N/A	
Autoimmune thyroiditis	1,092/34,820 (3.1)	196/23,235 (0.8)	3.81 (3.27-4.43)	<0.001*	1.63 (1.38-1.92)	<0.001*	N/A		1.56 (1.32-1.84)	<0.001*
Graves' disease	881/34,820 (2.5)	260/23,235 (1.1)	2.29 (2.00-2.64)	<0.001*	0.83 (0.71-0.98)	0.026*	N/A		0.79 (0.67-0.93)	0.005*
CCI ≥ 2 score (n = 28,692)										
Hypothyroidism	1,308/10,087 (13.0)	957/18,605 (5.1)	2.75 (2.52-3.00)	<0.001*	1.18 (1.06-1.31)	0.002*	1.15 (1.04-1.28)	0.009*	1.18 (1.06-1.31)	0.003*
Hyperthyroidism	792/10,087 (7.9)	585/18,605 (3.1)	2.62 (2.35-2.93)	<0.001*	1.02 (0.89-1.17)	0.759	1.00 (0.87-1.14)	0.992	N/A	
Thyroiditis	614/10,087 (6.1)	428/18,605 (2.3)	2.75 (2.43-3.12)	<0.001*	1.31 (1.13-1.52)	<0.001*	1.28 (1.11-1.49)	0.001*	N/A	
Autoimmune thyroiditis	277/10,087 (2.8)	191/18,605 (1.0)	2.72 (2.26-3.28)	<0.001*	1.07 (0.86-1.33)	0.543	N/A		1.03 (0.83-1.29)	0.795
Graves' disease	273/10,087 (2.7)	177/18,605 (1.0)	2.89 (2.39-3.50)	<0.001*	0.95 (0.75-1.21)	0.699	N/A		0.94 (0.74-1.19)	0.619
Total cholesterol < 200 mg/dL (n = 247,272)										
Hypothyroidism	15,333/125,559 (12.2)	5,176/121,713 (4.3)	3.13 (3.03-3.24)	<0.001*	1.30 (1.26-1.35)	<0.001*	1.29 (1.24-1.34)	<0.001*	1.31 (1.26-1.36)	<0.001*
Hyperthyroidism	9,204/125,559 (7.3)	3,834/121,713 (3.2)	2.43 (2.34-2.53)	<0.001*	0.84 (0.81-0.89)	<0.001*	0.80 (0.77-0.84)	<0.001*	N/A	
Thyroiditis	8,630/125,559 (6.9)	2,739/121,713 (2.3)	3.21 (3.07-3.35)	<0.001*	1.37 (1.31-1.44)	<0.001*	1.35 (1.28-1.42)	<0.001*	N/A	
Autoimmune thyroiditis	4,006/125,559 (3.2)	1,182/121,713 (1.0)	3.36 (3.15-3.59)	<0.001*	1.19 (1.11-1.28)	<0.001*	N/A		1.15 (1.06-1.23)	<0.001*
Graves' disease	3,439/125,559 (2.7)	1,313/121,713 (1.1)	2.58 (2.42-2.75)	<0.001*	0.75 (0.70-0.82)	<0.001*	N/A		0.73 (0.67-0.79)	<0.001*
Total cholesterol ≥ 200 to < 240 mg/dL (n = 129,491)										
Hypothyroidism	7,421/64,098 (11.6)	2,746/65,393 (4.2)	2.99 (2.86-3.13)	<0.001*	1.27 (1.20-1.34)	<0.001*	1.25 (1.19-1.32)	<0.001*	1.27 (1.20-1.34)	<0.001*
Hyperthyroidism	4,133/64,098 (6.5)	1,935/65,393 (3.0)	2.26 (2.14-2.39)	<0.001*	0.83 (0.78-0.89)	<0.001*	0.80 (0.74-0.85)	<0.001*	N/A	
Thyroiditis	4,085/64,098 (6.4)	1,392/65,393 (2.1)	3.13 (2.94-3.33)	<0.001*	1.41 (1.32-1.52)	<0.001*	1.39 (1.30-1.49)	<0.001*	N/A	

Autoimmune thyroiditis	1,916/64,098 (3.0)	609/65,393 (0.9)	3.28 (2.99-3.59)	<0.001*	1.24 (1.12-1.38)	<0.001*	N/A		1.20 (1.08-1.34)	0.001*
Graves' disease	1,446/64,098 (2.3)	660/65,393 (1.0)	2.26 (2.06-2.48)	<0.001*	0.69 (0.62-0.78)	<0.001*	N/A		0.67 (0.60-0.75)	<0.001*
Total cholesterol ≥ 240 mg/dL (n = 49,801)										
Hypothyroidism	2,712/23,625 (11.5)	1,072/26,176 (4.1)	3.04 (2.82-3.27)	<0.001*	1.33 (1.22-1.45)	<0.001*	1.32 (1.21-1.44)	<0.001*	1.34 (1.23-1.45)	<0.001*
Hyperthyroidism	1,370/23,625 (5.8)	713/26,176 (2.7)	2.20 (2.01-2.41)	<0.001*	0.83 (0.74-0.93)	0.001*	0.79 (0.70-0.88)	<0.001*	N/A	
Thyroiditis	1,426/23,625 (6.0)	516/26,176 (2.0)	3.19 (2.88-3.54)	<0.001*	1.38 (1.23-1.55)	<0.001*	1.36 (1.21-1.53)	<0.001*	N/A	
Autoimmune thyroiditis	640/23,625 (2.7)	231/26,176 (0.9)	3.12 (2.68-3.63)	<0.001*	1.26 (1.06-1.50)	0.008*	N/A		1.23 (1.03-1.46)	0.021*
Graves' disease	419/23,625 (1.8)	234/26,176 (0.9)	2.00 (1.70-2.35)	<0.001*	0.58 (0.48-0.71)	<0.001*	N/A		0.56 (0.46-0.69)	<0.001*
SBP < 140 mmHg and DBP < 90 mmHg (n = 370,946)										
Hypothyroidism	22,366/184,891 (12.1)	8,018/186,055 (4.3)	3.06 (2.98-3.14)	<0.001*	1.30 (1.26-1.34)	<0.001*	1.28 (1.25-1.32)	<0.001*	1.30 (1.26-1.34)	<0.001*
Hyperthyroidism	12,855/184,891 (7.0)	5,782/186,055 (3.1)	2.33 (2.26-2.40)	<0.001*	0.84 (0.81-0.87)	<0.001*	0.80 (0.77-0.83)	<0.001*	N/A	
Thyroiditis	12,617/184,891 (6.8)	4,246/186,055 (2.3)	3.14 (3.03-3.25)	<0.001*	1.37 (1.31-1.42)	<0.001*	1.35 (1.29-1.40)	<0.001*	N/A	
Autoimmune thyroiditis	5,885/184,891 (3.2)	1,845/186,055 (1.0)	3.28 (3.11-3.46)	<0.001*	1.22 (1.15-1.29)	<0.001*	N/A		1.17 (1.10-1.25)	<0.001*
Graves' disease	4,686/184,891 (2.5)	1,975/186,055 (1.1)	2.42 (2.30-2.55)	<0.001*	0.73 (0.68-0.78)	<0.001*	N/A		0.70 (0.66-0.75)	<0.001*
SBP ≥ 140 mmHg or DBP ≥ 90 mmHg (n = 55,618)										
Hypothyroidism	3,100/28,391 (10.9)	976/27,227 (3.6)	3.30 (3.06-3.55)	<0.001*	1.29 (1.19-1.41)	<0.001*	1.27 (1.17-1.39)	<0.001*	1.29 (1.19-1.41)	<0.001*
Hyperthyroidism	1,852/28,391 (6.5)	700/27,227 (2.6)	2.65 (2.42-2.89)	<0.001*	0.85 (0.76-0.95)	0.003*	0.82 (0.73-0.91)	<0.001*	N/A	
Thyroiditis	1,524/28,391 (5.4)	401/27,227 (1.5)	3.80 (3.40-4.24)	<0.001*	1.58 (1.40-1.79)	<0.001*	1.55 (1.37-1.76)	<0.001*	N/A	
Autoimmune thyroiditis	677/28,391 (2.4)	177/27,227 (0.7)	3.73 (3.16-4.41)	<0.001*	1.23 (1.02-1.49)	0.030*	N/A		1.19 (0.98-1.44)	0.076

Graves' disease	618/28,391 (2.2)	232/27,227 (0.9)	2.59 (2.22-3.01)	<0.001*	0.65 (0.54-0.79)	<0.001*	N/A		0.63 (0.52-0.77)	<0.001*
Fasting blood glucose < 100 mg/dL (n = 306,614)										
Hypothyroidism	18,856/153,297 (12.3)	6,530/153,317 (4.3)	3.15 (3.06-3.25)	<0.001*	1.30 (1.26-1.35)	<0.001*	1.29 (1.25-1.33)	<0.001*	1.30 (1.26-1.35)	<0.001*
Hyperthyroidism	10,654/153,297 (7.0)	4,645/153,317 (3.0)	2.39 (2.31-2.48)	<0.001*	0.84 (0.80-0.88)	<0.001*	0.80 (0.76-0.83)	<0.001*	N/A	
Thyroiditis	10,650/153,297 (7.0)	3,442/153,317 (2.3)	3.25 (3.13-3.38)	<0.001*	1.37 (1.31-1.44)	<0.001*	1.35 (1.29-1.41)	<0.001*	N/A	
Autoimmune thyroiditis	5,006/153,297 (3.3)	1,518/153,317 (1.0)	3.38 (3.19-3.58)	<0.001*	1.19 (1.11-1.27)	<0.001*	N/A		1.14 (1.07-1.22)	<0.001*
Graves' disease	3,882/153,297 (2.5)	1,600/153,317 (1.0)	2.46 (2.32-2.61)	<0.001*	0.73 (0.68-0.79)	<0.001*	N/A		0.71 (0.66-0.76)	<0.001*
Fasting blood glucose ≥ 100 mg/dL (n = 119,950)										
Hypothyroidism	6,610/59,985 (11.0)	2,464/59,965 (4.1)	2.89 (2.76-3.03)	<0.001*	2.89 (2.76-3.03)	<0.001*	1.26 (1.19-1.33)	<0.001*	1.27 (1.20-1.34)	<0.001*
Hyperthyroidism	4,053/59,985 (6.8)	1,837/59,965 (3.1)	2.29 (2.17-2.43)	<0.001*	2.29 (2.17-2.43)	<0.001*	0.80 (0.75-0.86)	<0.001*	N/A	
Thyroiditis	3,491/59,985 (5.8)	1,205/59,965 (2.0)	3.01 (2.82-3.22)	<0.001*	3.01 (2.82-3.22)	<0.001*	1.38 (1.28-1.48)	<0.001*	N/A	
Autoimmune thyroiditis	1,556/59,985 (2.6)	504/59,965 (0.8)	3.14 (2.84-3.48)	<0.001*	3.14 (2.84-3.48)	<0.001*	N/A		1.24 (1.11-1.39)	<0.001*
Graves' disease	1,422/59,985 (2.4)	607/59,965 (1.0)	2.37 (2.16-2.61)	<0.001*	2.37 (2.16-2.61)	<0.001*	N/A		0.65 (0.58-0.73)	<0.001*

* Un-conditional logistic regression, Significance at $P < 0.05$. † Model 1 was adjusted for age, sex, income, region of residence, total cholesterol, systolic blood pressure, diastolic blood pressure, fasting blood glucose, obesity, smoking, alcohol consumption, Charlson comorbidity index scores, and the number of thyroid function tests. ‡ Model 2 was adjusted for model 1 plus hypothyroidism, hyperthyroidism, and thyroiditis. § Model 3 was adjusted for model 1 plus hypothyroidism, autoimmune thyroiditis, and Graves' disease.
